# Supplementary material for: Evolutionary conservation of the grape sex-determining region in angiosperms and emergence of dioecy in Vitaceae
Source: Nat Commun. 2025 Jul 1;16:6047. doi: 10.1038/s41467-025-61387-9 (PMC12215612; doi:10.1038/s41467-025-61387-9)
Supplement: Supplementary file 1 — Supplementary Information [file 41467_2025_61387_MOESM1_ESM.pdf]

Evolutionary conservation of the grape sex-determining region in angiosperms and emergence of dioecy in Vitaceae

Massonnet *et al.*

Supplementary Table 1. Cumulative length of the repetitive element classes in the muscadine SDR. Source data are provided as a Source Data file.

**Repeat class**	**Fry**		**DVIT1750**		**Trayshed**	
	**Haplotype F1**	**Haplotype F2**	**Haplotype M**	**Haplotype F**	**Haplotype M**	**Haplotype F**
DNA/CMC-EnSpm	121	327	121	1,797	1,286	217
DNA/CMC-Transib	0	0	0	115	115	0
DNA/En-Spm	0	0	0	899	897	0
DNA/hAT-Ac	818	793	817	0	0	818
DNA/hAT-Tag1	841	852	841	1,737	1,737	653
DNA/MuDR	0	0	0	6,703	6,706	0
DNA/MULE-MuDR	1,589	1,650	1,589	11,218	11,433	1,269
DNA/PIF-Harbinger	3,136	3,572	3,144	2,235	2,023	2,945
Low_complexity	796	765	796	468	470	702
LTR/Caulimovirus	124	279	124	520	520	124
LTR/Copia	2,853	2,730	2,853	6,751	6,834	2,729
LTR/Custom	1,262	2,180	1,262	4,423	3,383	1,604
LTR/Gypsy	4,413	4,248	4,263	15,411	14,785	4,391
RC/Helitron	0	0	0	0	0	216
Simple_repeat	1,958	1,844	1,953	2,992	2,860	1,939
Unknown	781	602	781	2,546	2,546	788
Unspecified	4,214	4,477	4,214	47,311	46,732	6,113
Total	22,906	24,319	22,758	105,126	102,327	24,508

Supplementary Table 2. Coordinates of the ends of the inversion between the F and M haplotypes in *M. rotundifolia* Trayshed and DVIT1750. Source data are provided as a Source Data file.

**Accession**	**F haplotype**			**M haplotype**		
	**Contig**	**Start**	**Stop**	**Contig**	**Start**	**Stop**
Trayshed	hap2.chr02	4,269,845	4,277,860	hap1.chr02	4,715,941	4,707,885
	hap2.chr02	4,408,648	4,409,128	hap1.chr02	4,507,417	4,506,939
DVIT1750	primary000004F	4,318,393	4,326,409	haplotig000004F_009	582,364	574,306
	primary000004F	4,454,155	4,454,777	haplotig000004F_009	373,984	373,347

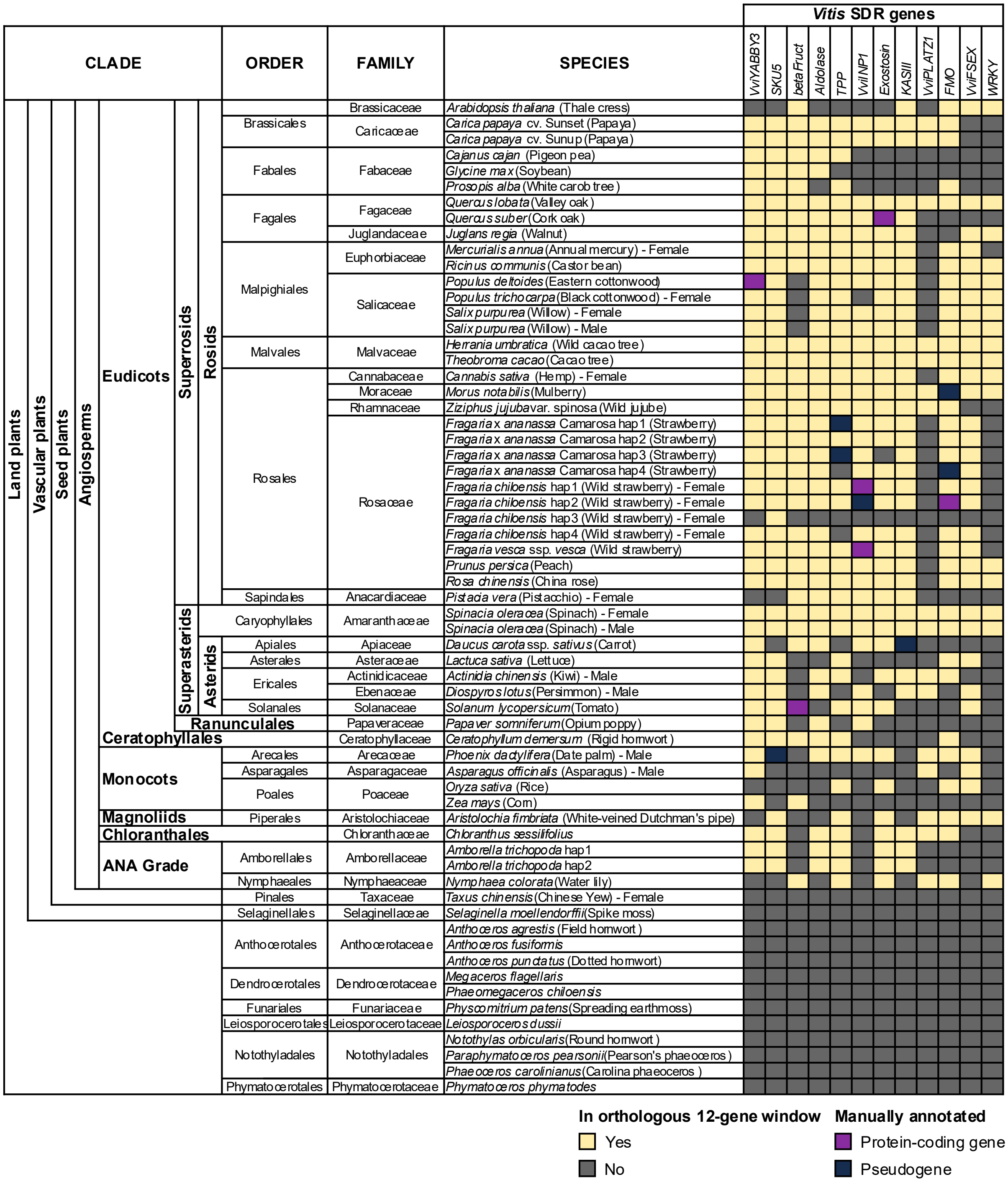

Supplementary Fig. 1. Gene content of the 12-gene window orthologous to Cabernet Sauvignon sex-determining region (haplotype 1) among 56 plant genomes. Source data are provided as a Source Data file.

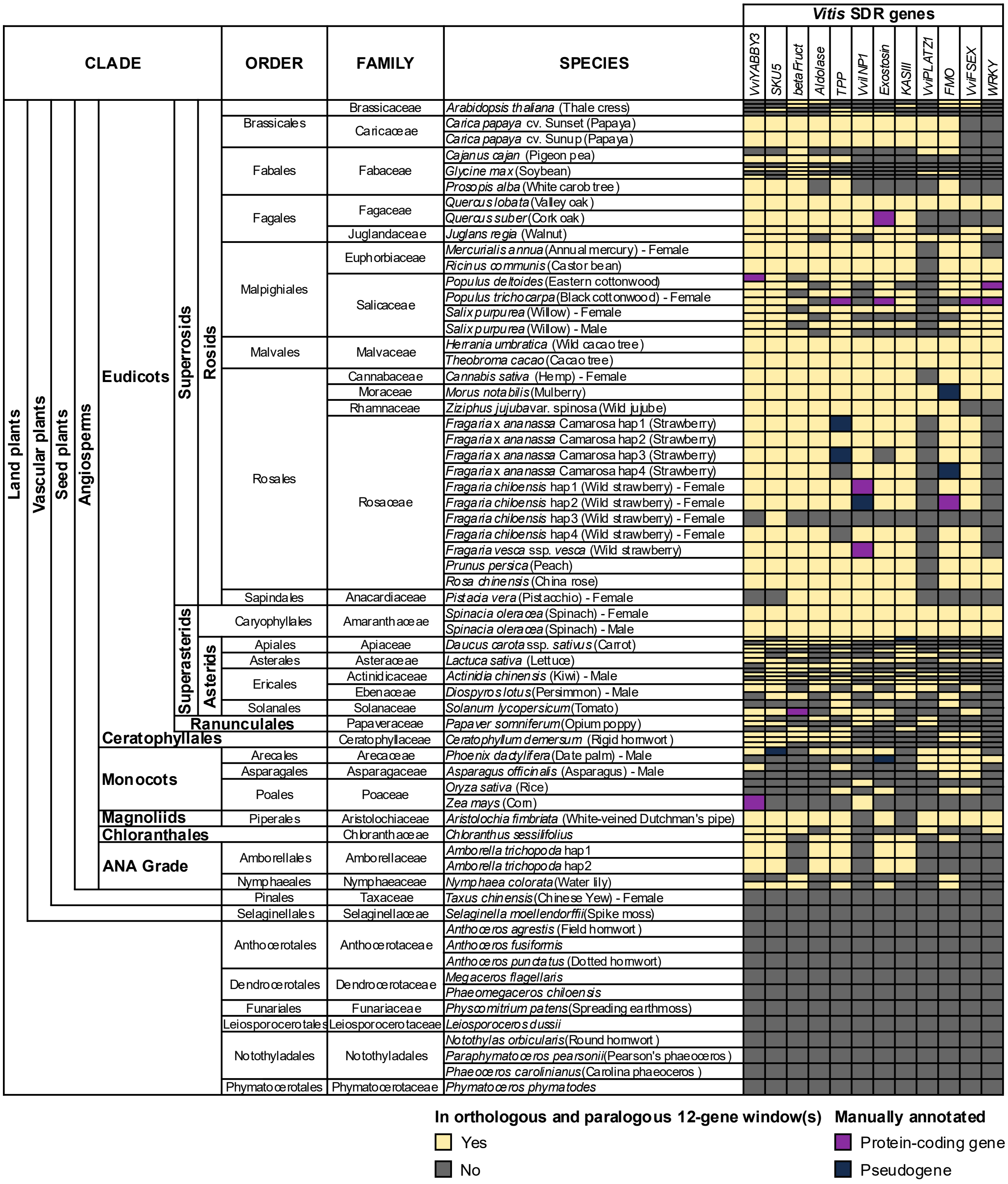

Supplementary Fig. 2. Gene content of the 12-gene window orthologous and paralogous to Cabernet Sauvignon sex-determining region (haplotype 1) among 56 plant genomes. Source data are provided as a Source Data file.

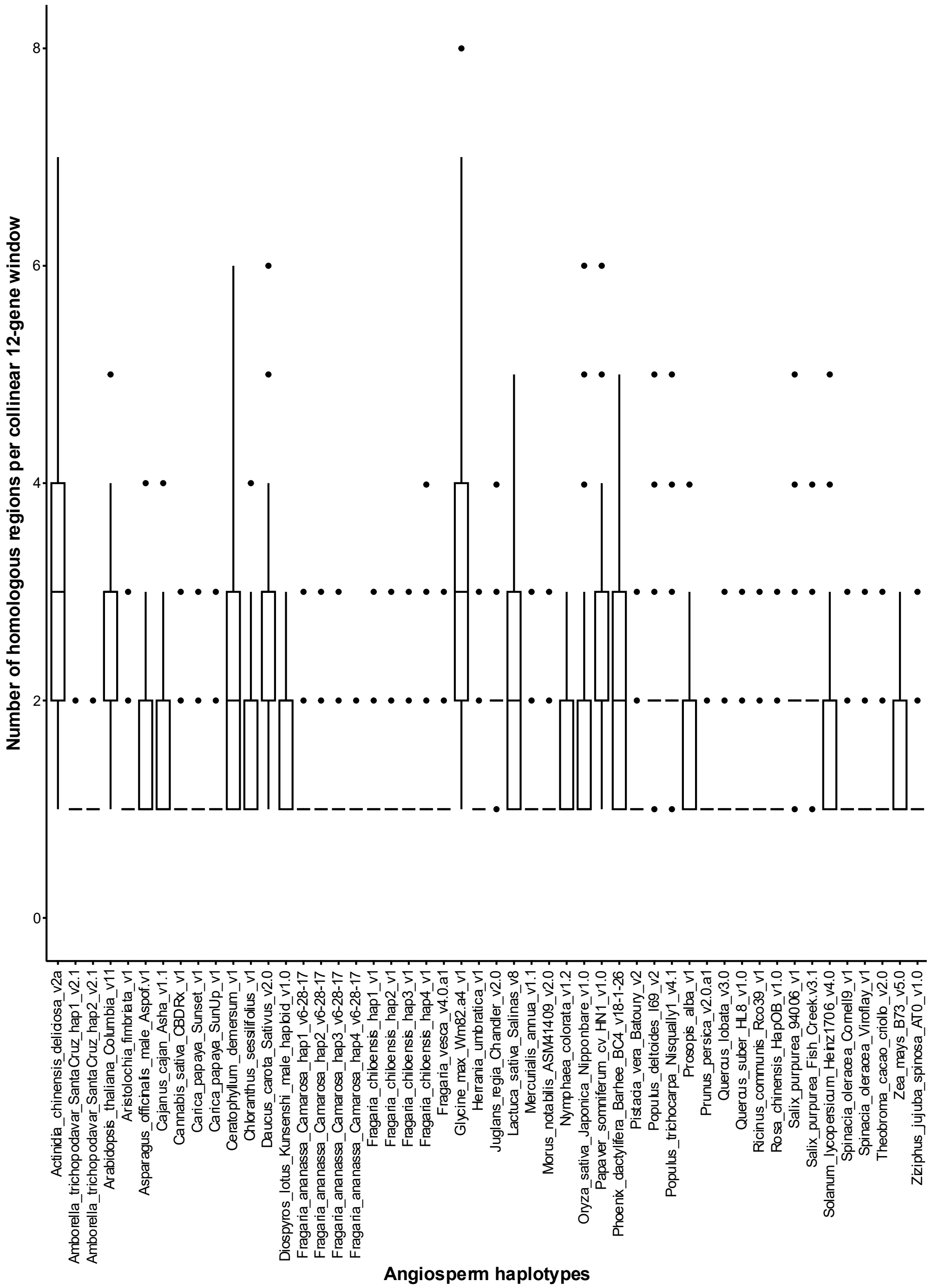

Supplementary Fig. 3. Number of homologous regions to each 12-gene window of the haplotype 1 of Cabernet Sauvignon genome among 50 angiosperm genome haplotypes. The middle bars represent the median, while the bottom and top of each box represent the 25th and 75th percentiles, respectively, and the whiskers extend to 1.5 times the interquartile range. Dots are outliers. Source data are provided as a Source Data file.

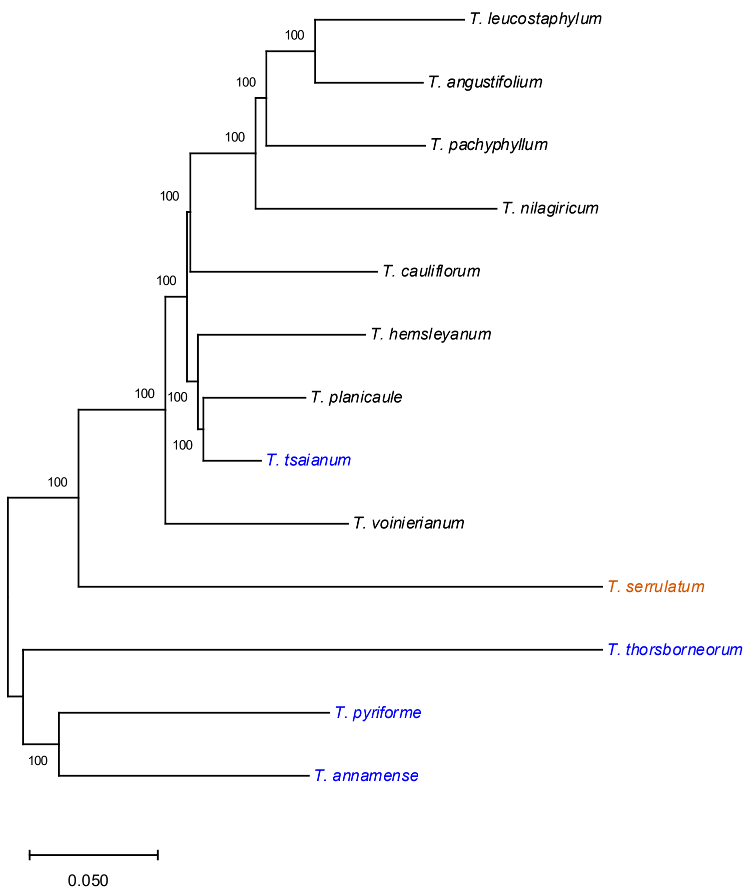

Supplementary Fig. 4. Phylogenetic tree of the thirteen *Tetrastigma* species used for evaluating the linkage disequilibrium in the genomic region homologous of the *Vitis* sex-determining region. Species written in orange, blue, and black were identified as part of the *Tetrastigma* clades IV, V, VI, respectively, using 10 chloroplast loci (Peng *et al*.[1]). Scale bar is in the unit of the number of substitutions per site. Source data are provided as a Source Data file.

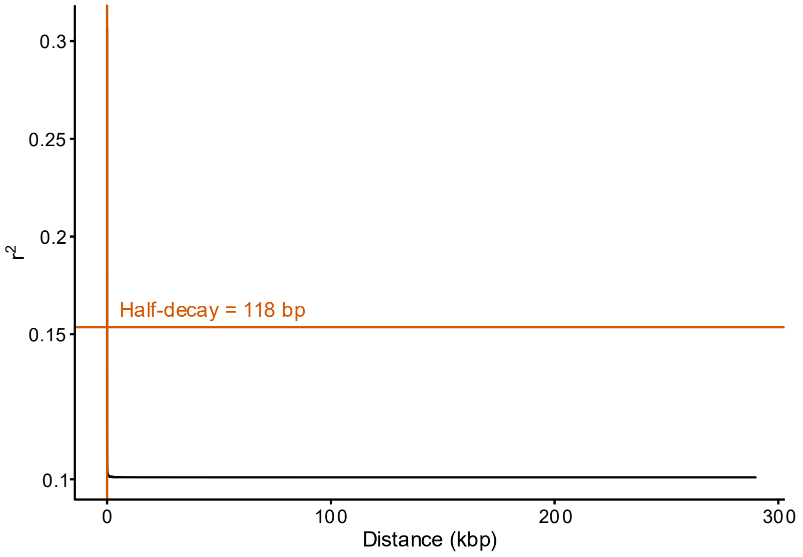

Supplementary Fig. 5. Linkage disequilibrium decay in the SNP dataset from the alignment of the whole-genome sequencing of thirteen *Tetrastigma* species on *T. voinieranum* haplotype 1. Source data are provided as a Source Data file.

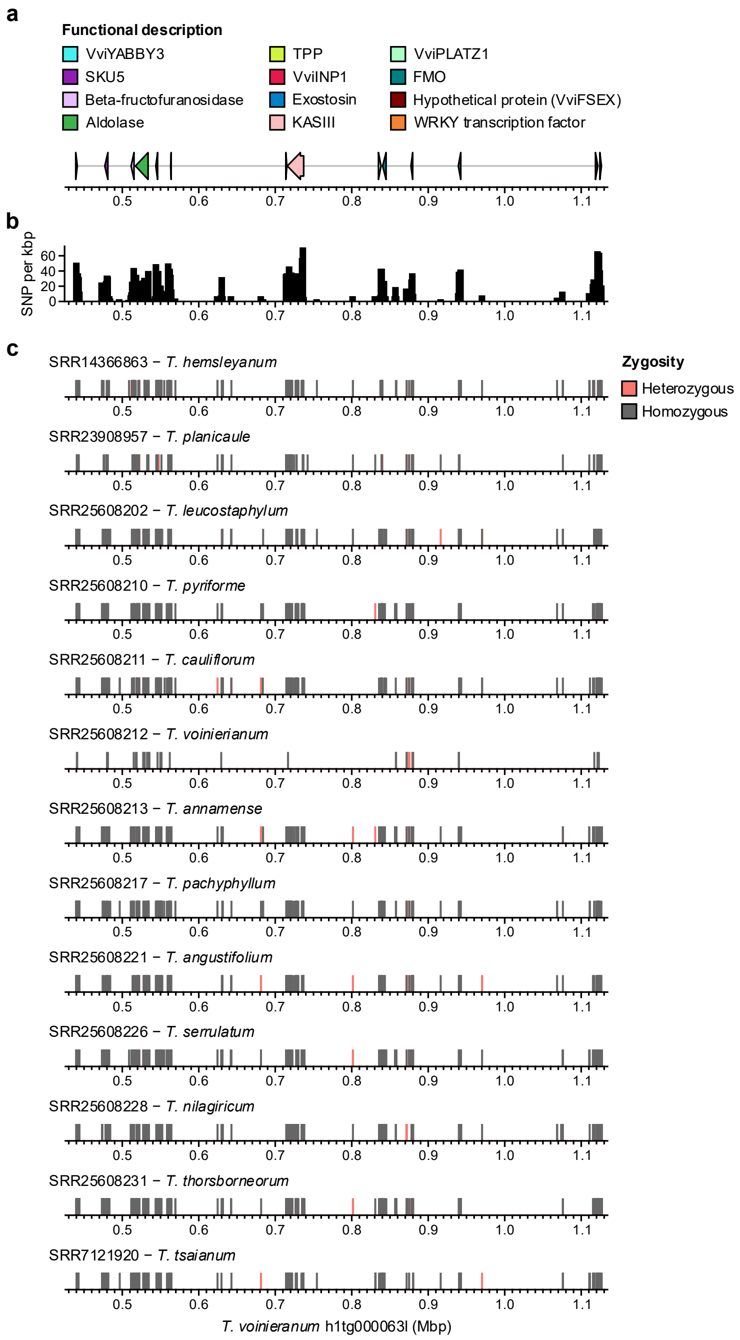

Supplementary Fig. 6. Distribution of the SNPs identified in thirteen *Tetrastigma* species compared to the homologous region of the *Vitis* sex-determining region of *T. voinieranum*. (**a**) Schematic representation of the gene content of the homologous region of the *Vitis* sex-determining region in the Haplotype 1 of *T. voinieranum*. (**b**) Number of SNPs per window of 1 kbp. (**c**) Location of the SNPs detected in each *Tetrastigma* species compared to the homologous region of the *Vitis* sex-determining region of *T. voinieranum*. Source data are provided as a Source Data file.

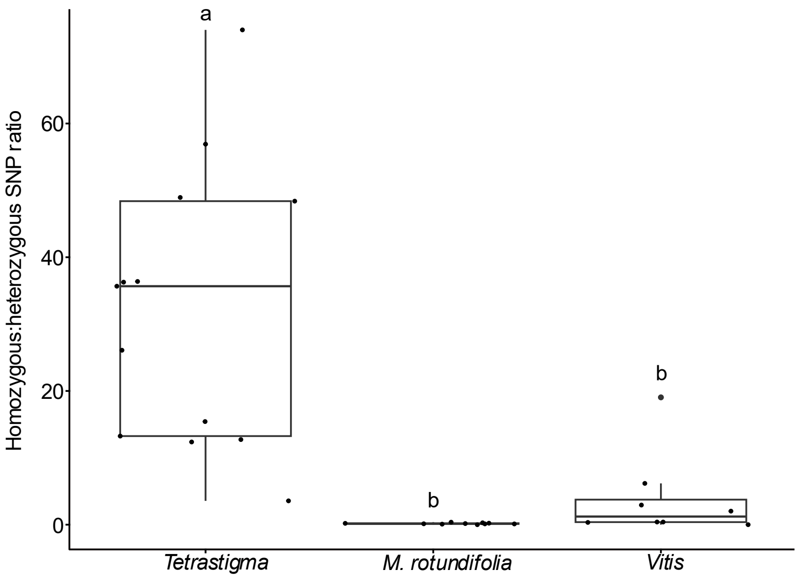

Supplementary Fig. 7. Ratio between the number of homozygous and heterozygous SNPs in the homologous region of the *Vitis* sex-determining region (SDR) in thirteen *Tetrastigma* species, in the muscadine SDR in eleven *M. rotundifolia* accessions, and in the *Vitis* SDR in eight *Vitis* species. The middle bars represent the median, while the bottom and top of each box represent the 25th and 75th percentiles, respectively, and the whiskers extend to 1.5 times the interquartile range. Dots are outliers. Significant differences between genera are indicated by different letters (Kruskal-Wallis test followed by post hoc Dunn's test; P value < 0.05). On average, 326.6 ± 176.2 SNPs were found in the thirteen species of *Tetrastigma*, while 2,602.5 ± 1,203.4 SNPs were found in ten muscadines compared to the haplotype 2 of *M. rotundifolia* Trayshed, and 1,316.6 ± 486.4 SNPs were identified in eight *Vitis* species compared to the haplotype 2 of Cabernet Sauvignon. Source data are provided as a Source Data file.

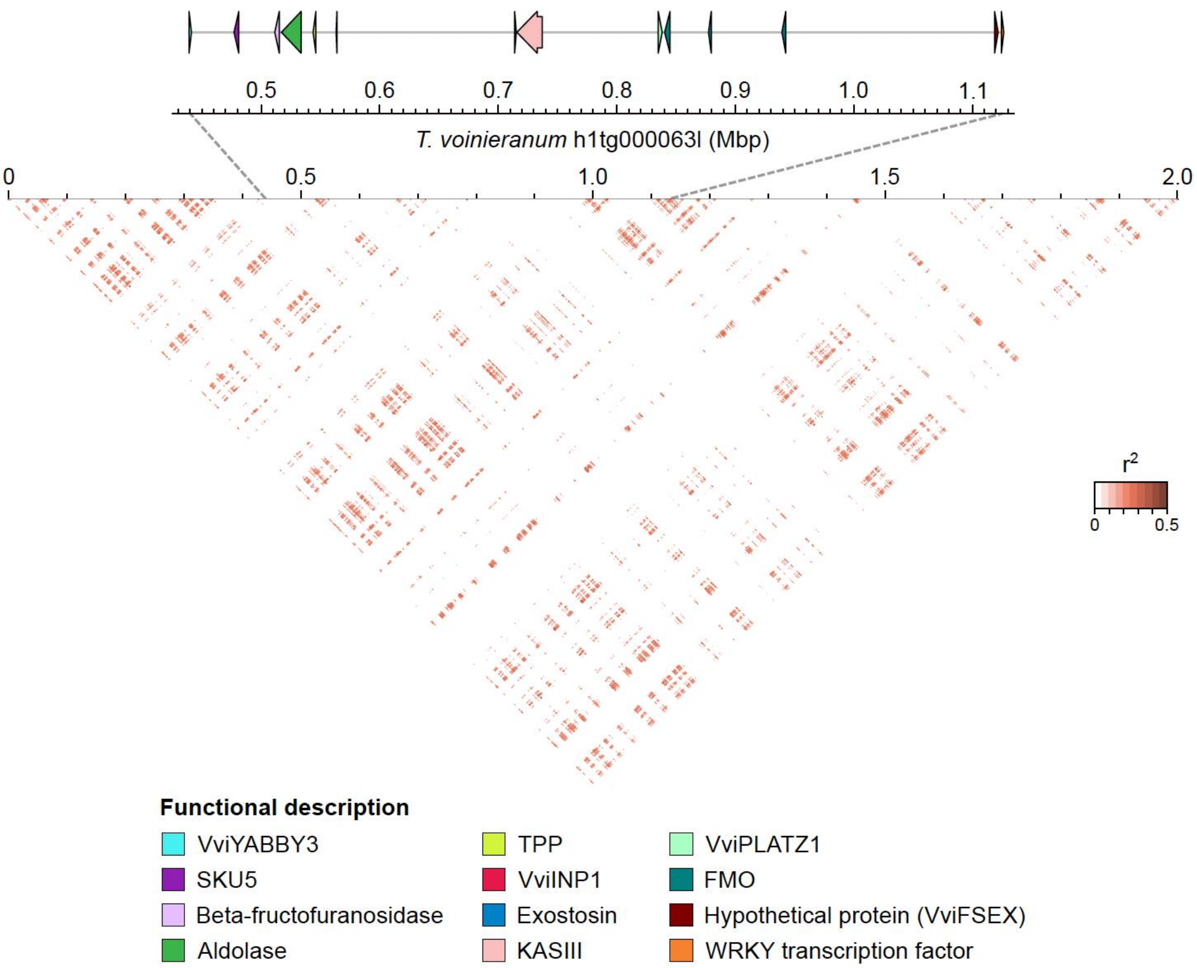

Supplementary Fig. 8. Linkage disequilibrium in the 2-Mbp region around the homologous region of the *Vitis* sex-determining region in *T. voinieranum* as the mean r^2 per kbp^2. Source data are provided as a Source Data file.

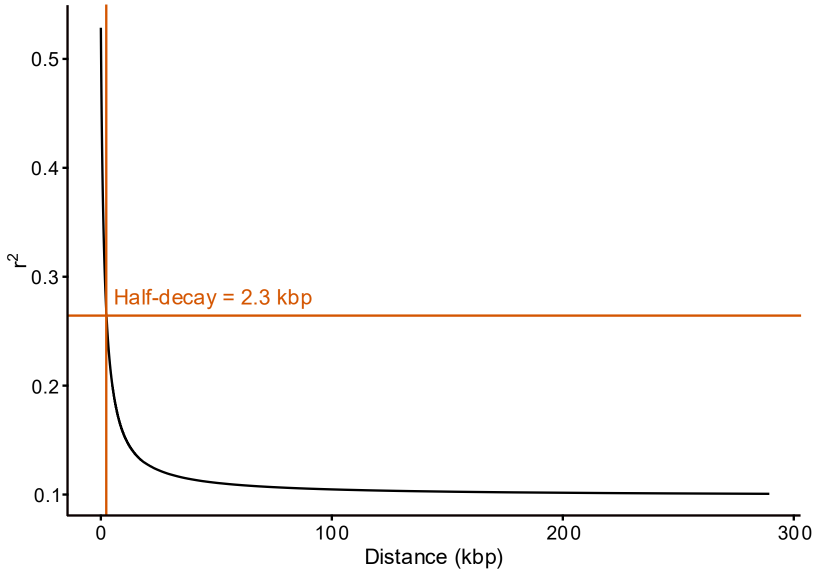

Supplementary Fig. 9. Linkage disequilibrium decay in the SNP dataset from the alignment of the whole-genome sequencing of ten *M. rotundifolia* accessions on *M. rotundifolia* Trayshed haplotype 2. Source data are provided as a Source Data file.

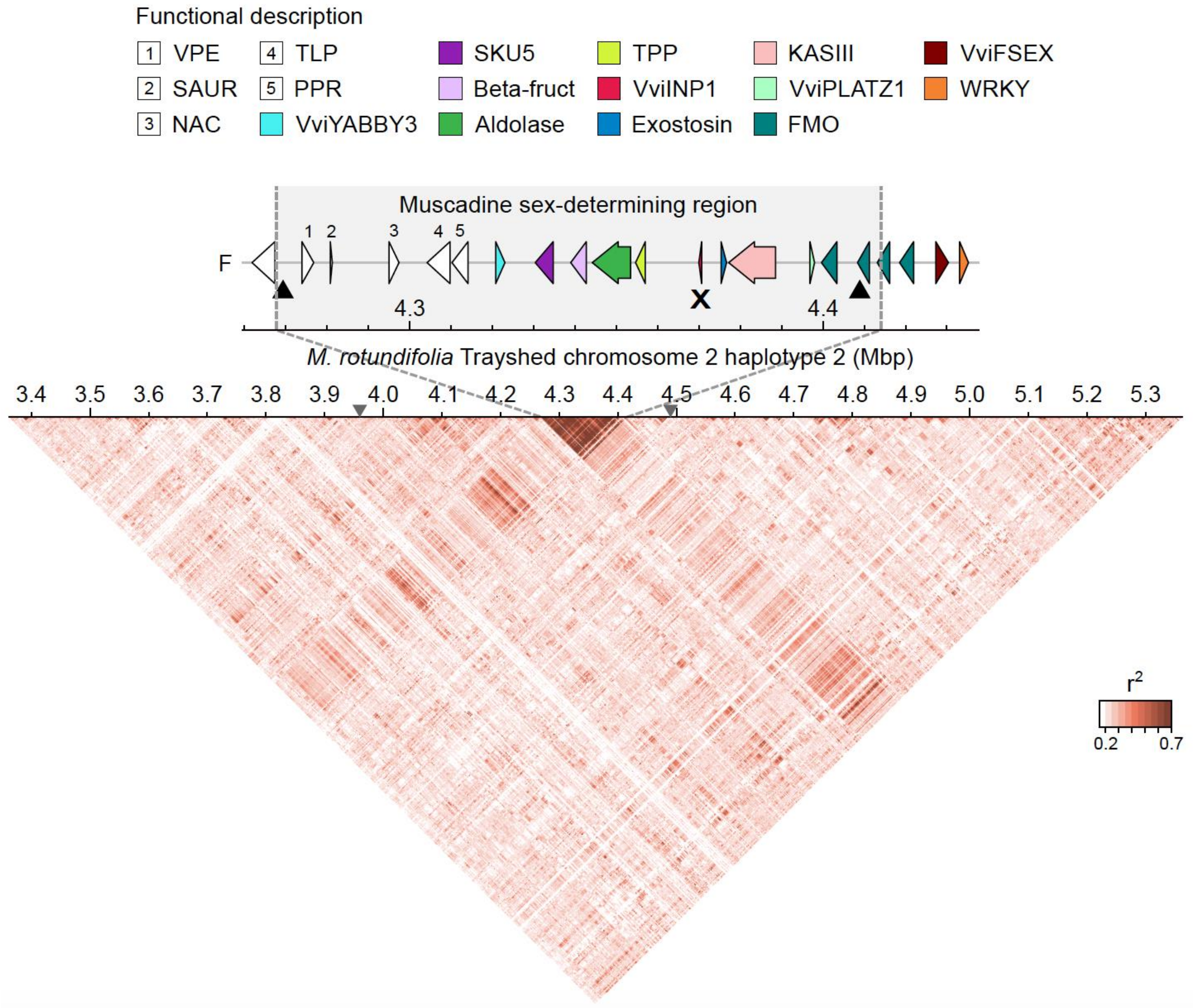

Supplementary Fig. 10. Linkage disequilibrium in the 2-Mbp region around the *M. rotundifolia* sex-determining region as the mean r^2 per kbp[2]. The gray triangles on the scale mark the position of the sex-linked markers in muscadines, S2_4635912 and S2_5085983 (Lewter *et al*.[2]). White arrows depict genes that are not sex-linked in *Vitis* spp. The black triangles on the scale mark the position of the breaking points of the inversion in the M haplotype compared to the F haplotype. Pseudogenes are indicated with an X. Source data are provided as a Source Data file.

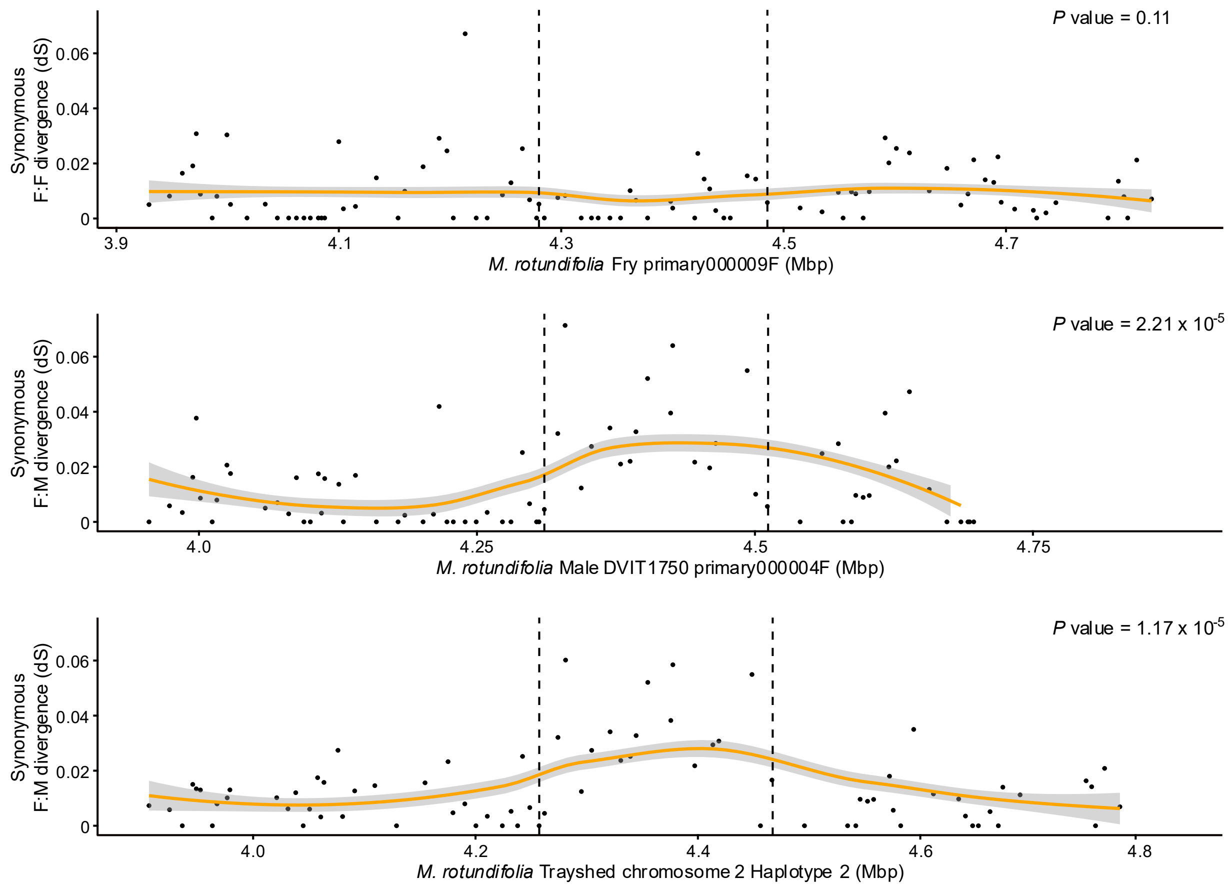

Supplementary Fig. 11. Synonymous divergence (dS) between the two haplotypes containing the sex-determining region of the *M. rotundifolia* Fry (female), DVIT1750 (male), and Trayshed (male). Difference of dS within and outside the sex-determining region was tested using a Kruskal-Wallis test. The orange line represents the loess-smoothed segmented linear model of the dS with a 95% confidence interval. Source data are provided as a Source Data file.

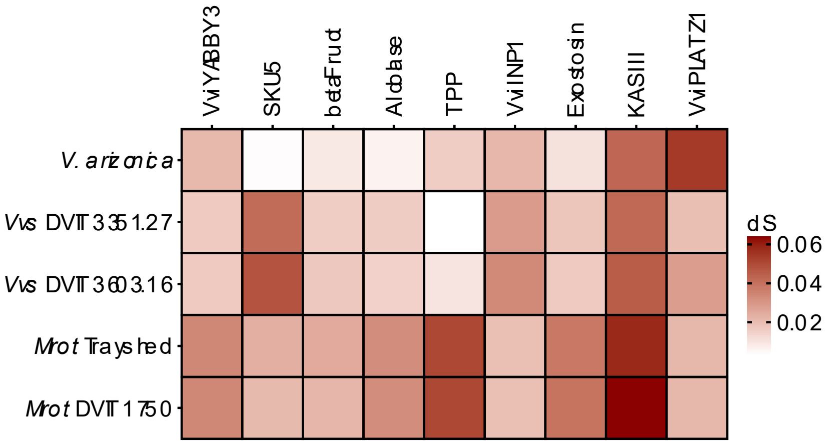

Supplementary Fig. 12. Synonymous divergence (dS) between the F-associated and M-associated haplotypes in the sex-determining region in the males *V. arizonica* b40-14, *V. vinifera* ssp. *sylvestris* (*Vvs*) DVIT3351.27 and DVIT3603.16, and the *Muscadinia rotundifolia* (*Mrot*) Trayshed and DVIT1750. Source data are provided as a Source Data file.

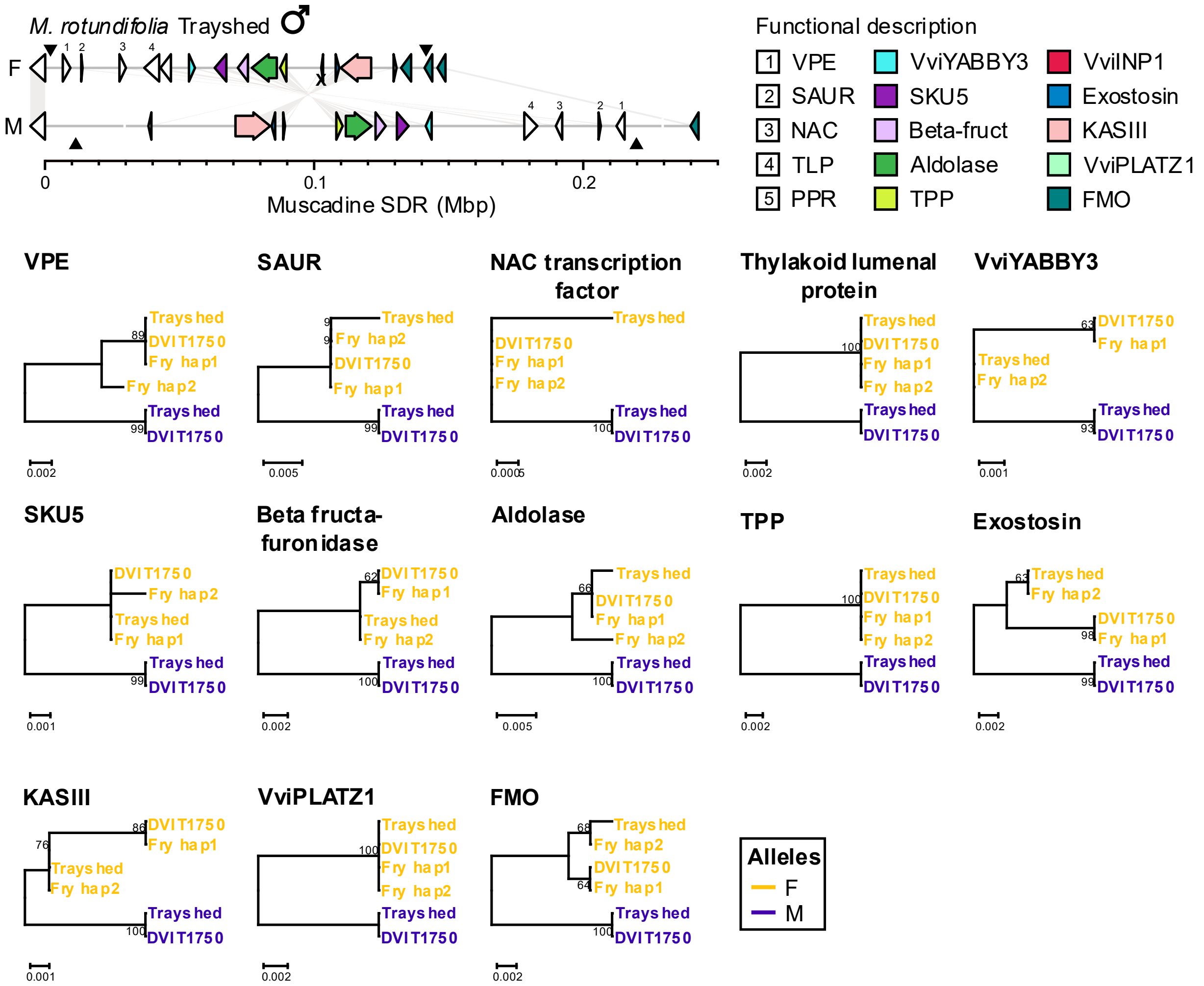

Supplementary Fig. 13. Neighbor-joining clustering of the protein sequences encoded by each gene of the sex-determining region in *Muscadinia rotundifolia*. Scale bars are in the unit of the number of substitutions per site. Abbreviations: hap, haplotype. Source data are provided as a Source Data file.

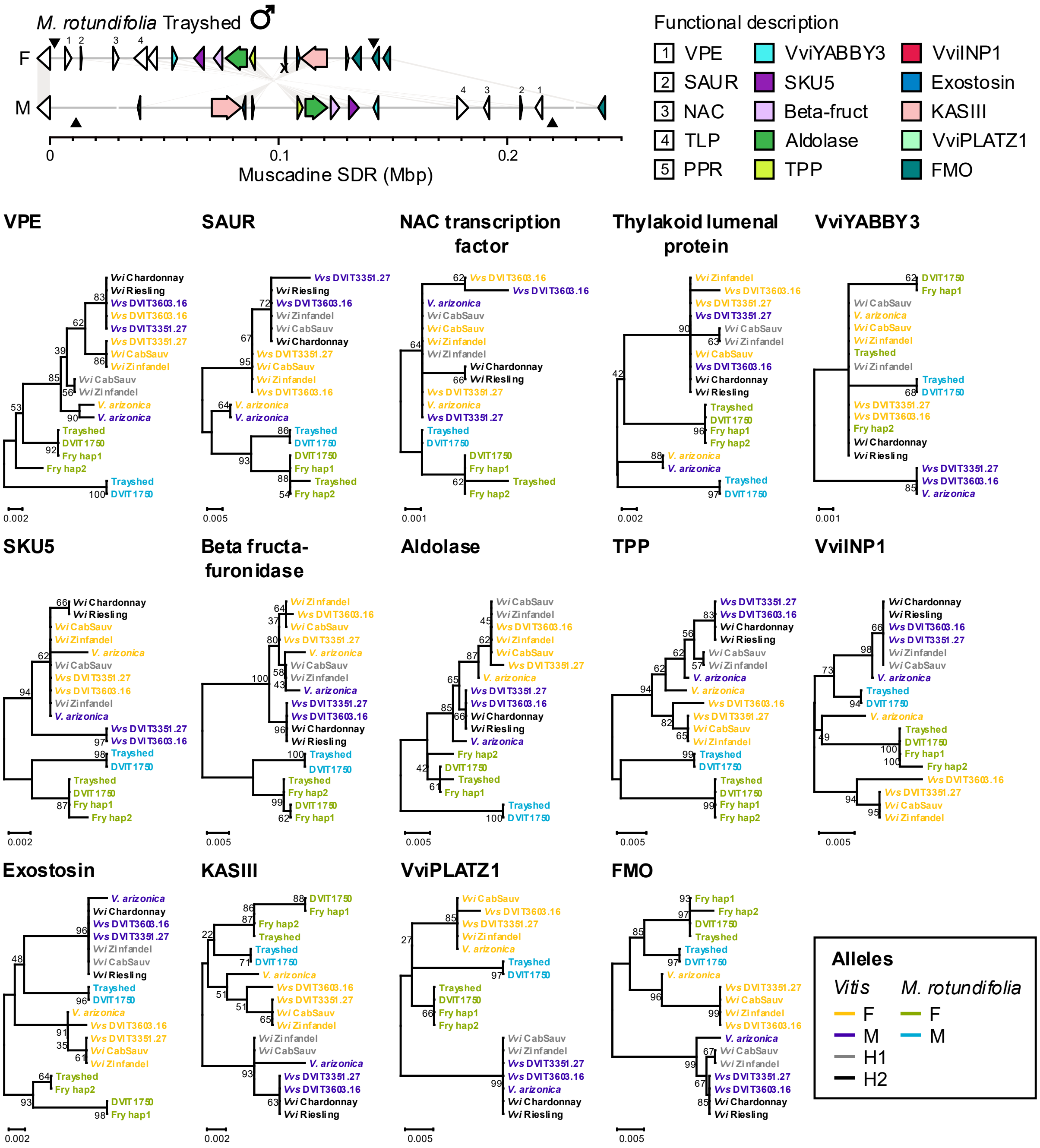

Supplementary Fig. 14. Neighbor-joining clustering of the protein sequences encoded by each gene of the sex-determining region in *Muscadinia rotundifolia*, *Vitis arizonica*, *V. vinifera* ssp. *sylvestris* (*Vvs*), *V. vinifera* ssp. *vinifera* (*Vvi*). Scale bars are in the unit of the number of substitutions per site. Abbreviations: CabSauv, Cabernet Sauvignon; hap, haplotype. Source data are provided as a Source Data file.

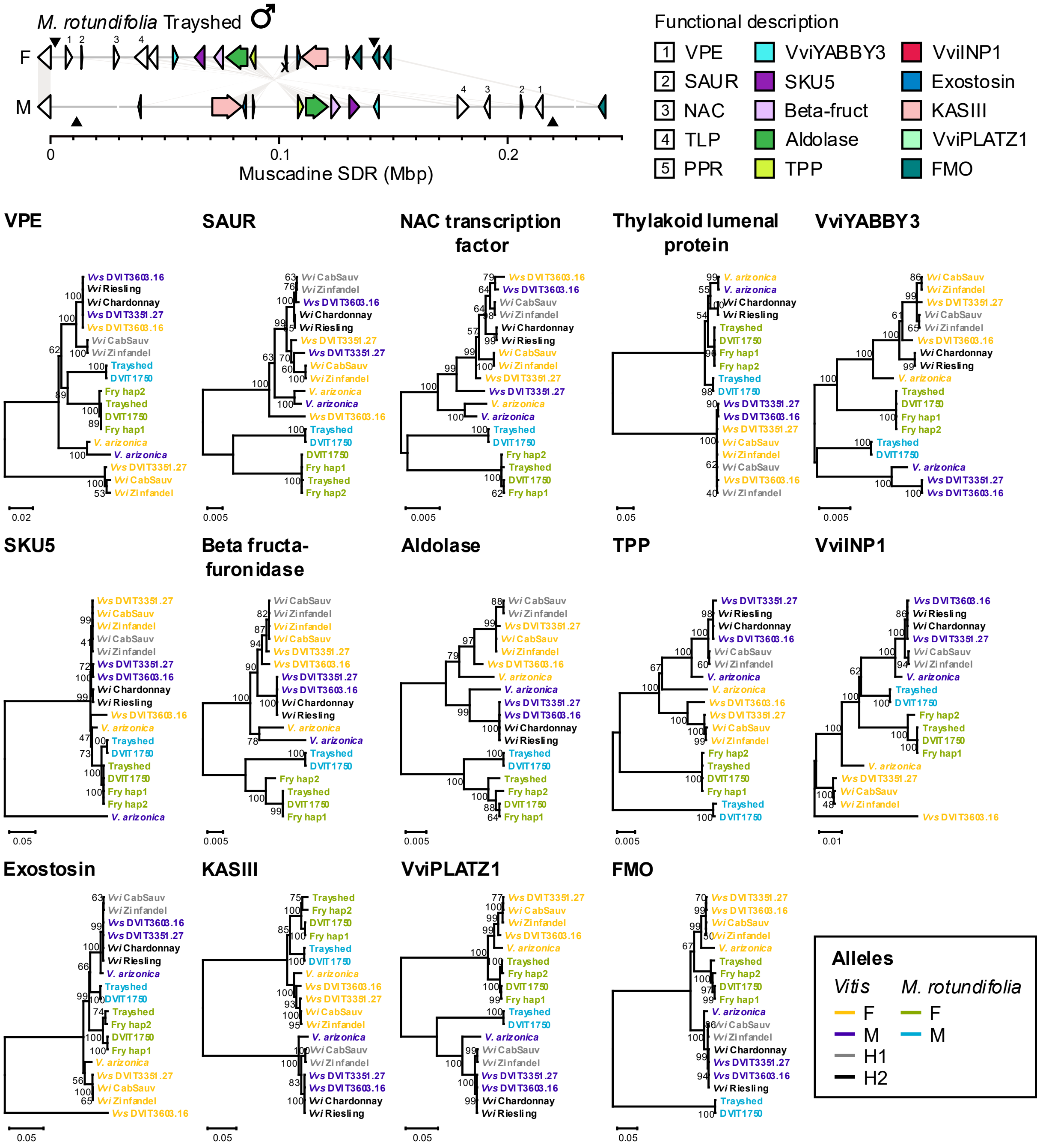

Supplementary Fig. 15. Neighbor-joining clustering of the promoter region (3-kbp region upstream of the transcription start site) of each gene of the sex-determining region in *Muscadinia rotundifolia*, *Vitis arizonica*, *V. vinifera* ssp. *sylvestris* (*Vvs*), *V. vinifera* ssp. *vinifera* (*Vvi*). Scale bars are in the unit of the number of substitutions per site. Abbreviations: CabSauv, Cabernet Sauvignon; hap, haplotype. Source data are provided as a Source Data file.

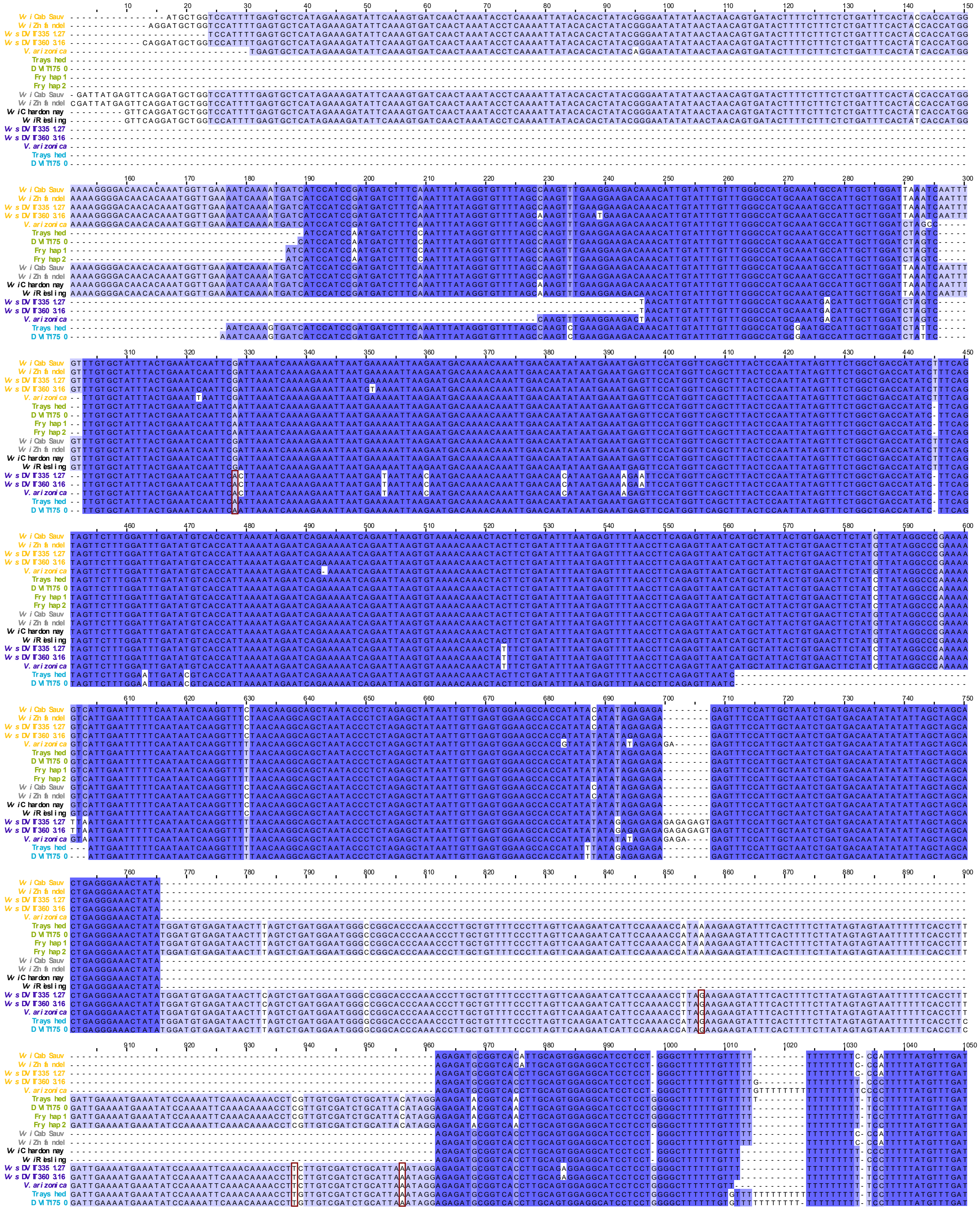

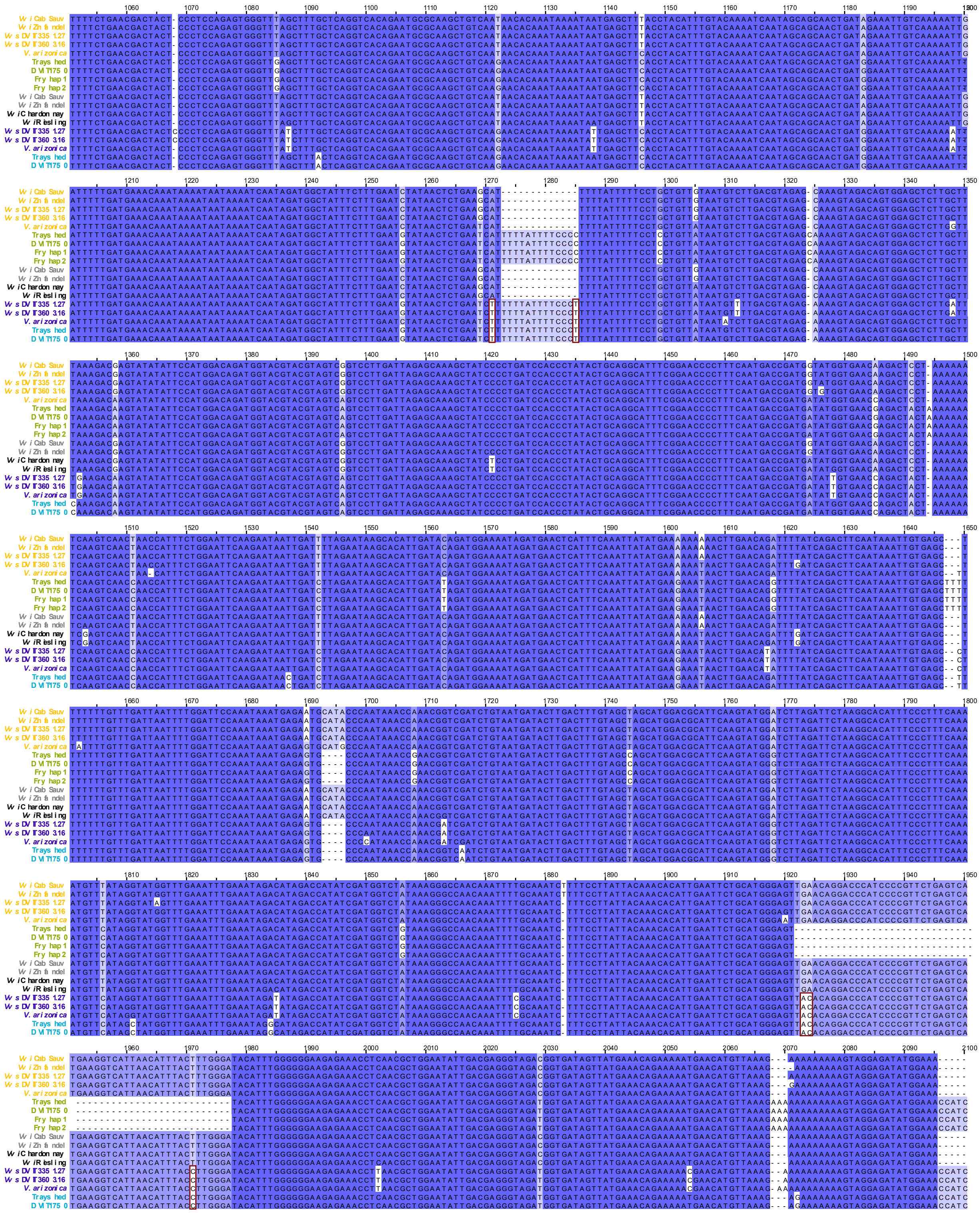

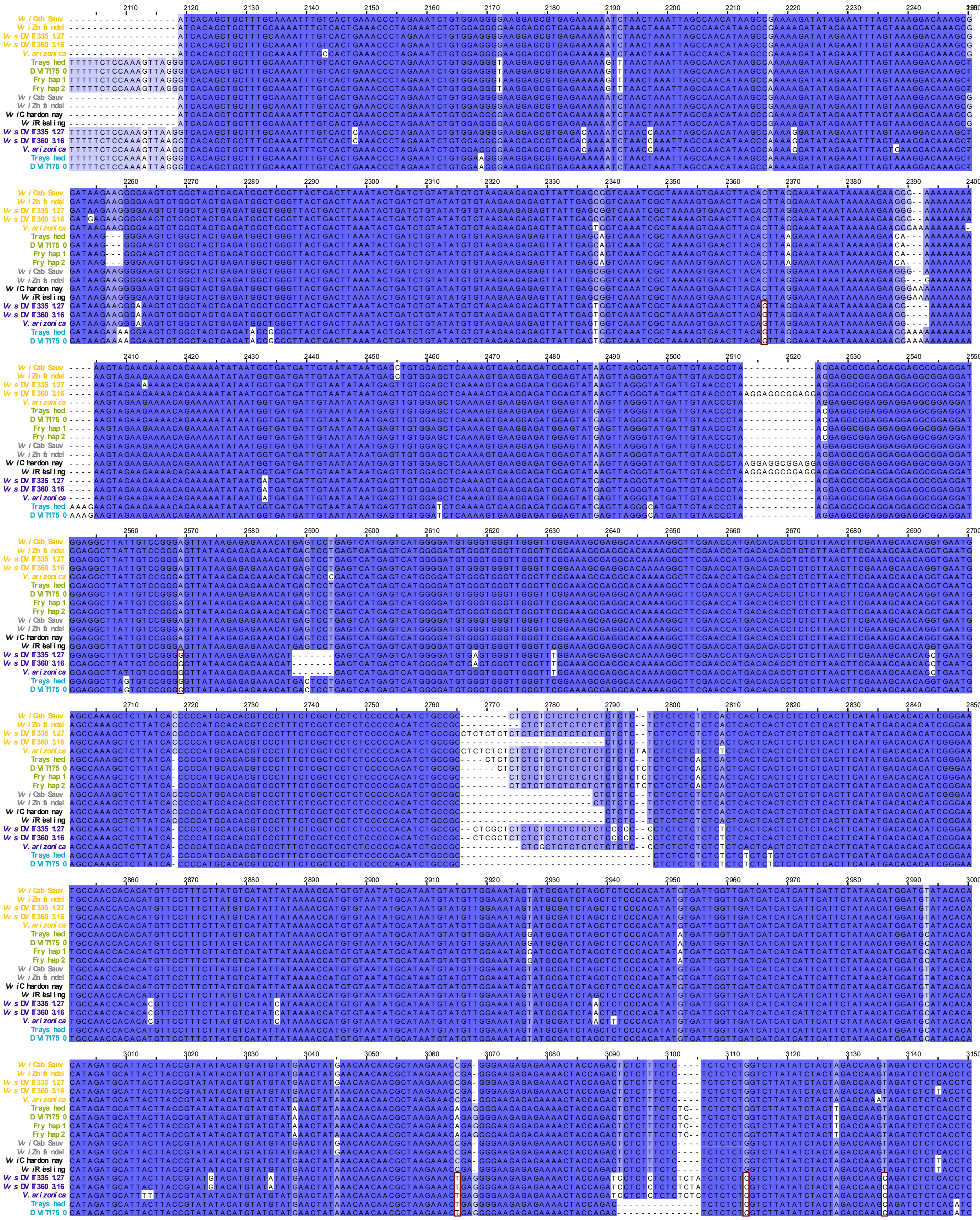

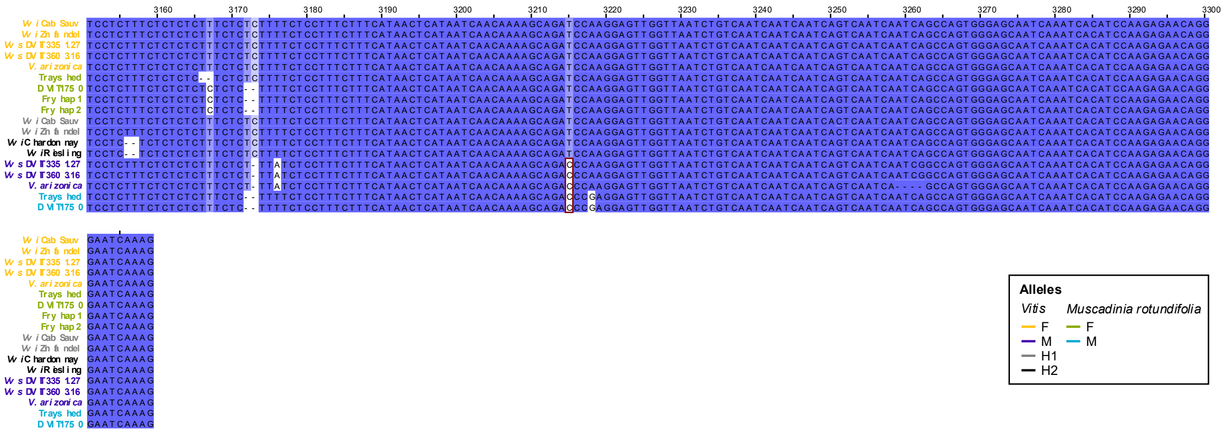

Supplementary Fig. 16. Sequence alignment of the promoter region (3-kbp region upstream of the transcription start site) of *VviYABBY3* in *Muscadinia rotundifolia*, *Vitis arizonica*, *V. vinifera* ssp. *sylvestris* (*Vvs*), *V. vinifera* ssp. *vinifera* (*Vvi*). M-specific sites are indicated by a red square. Abbreviations: CabSauv, Cabernet Sauvignon. Source data are provided as a Source Data file.

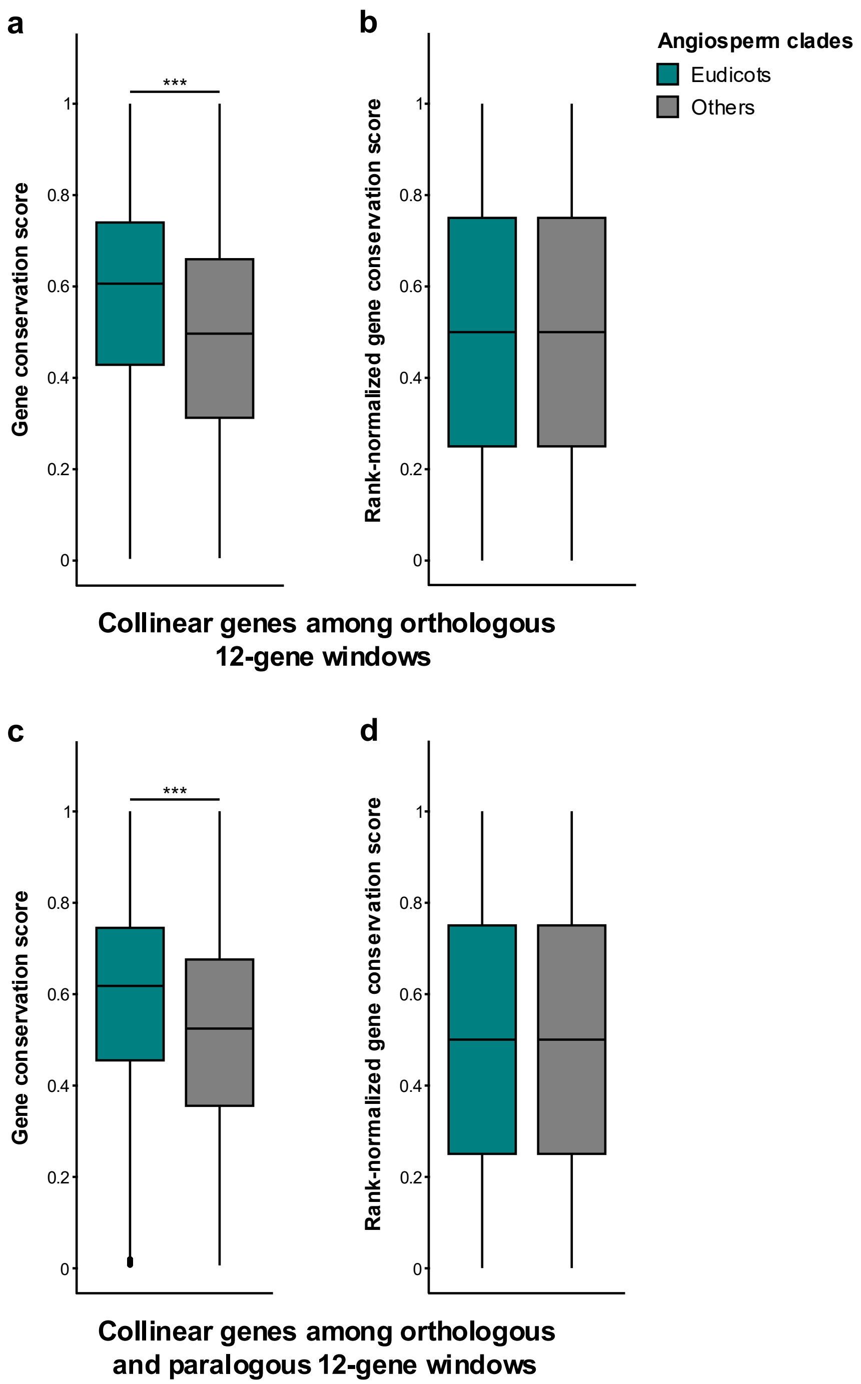

Supplementary Fig. 17. Distribution of the gene conservation scores of the collinear genes among orthologous (a,b) and orthologous and paralogous 12-gene windows (c,d), before (a,c) and after (b,d) rank-normalization. The middle bars represent the median, while the bottom and top of each box represent the 25th and 75th percentiles, respectively, and the whiskers extend to 1.5 times the interquartile range. Dots are outliers. Differences between angiosperm clades are indicated by different letters (Kruskal-Wallis test; $P \leq 0.0001$). Source data are provided as a Source Data file.

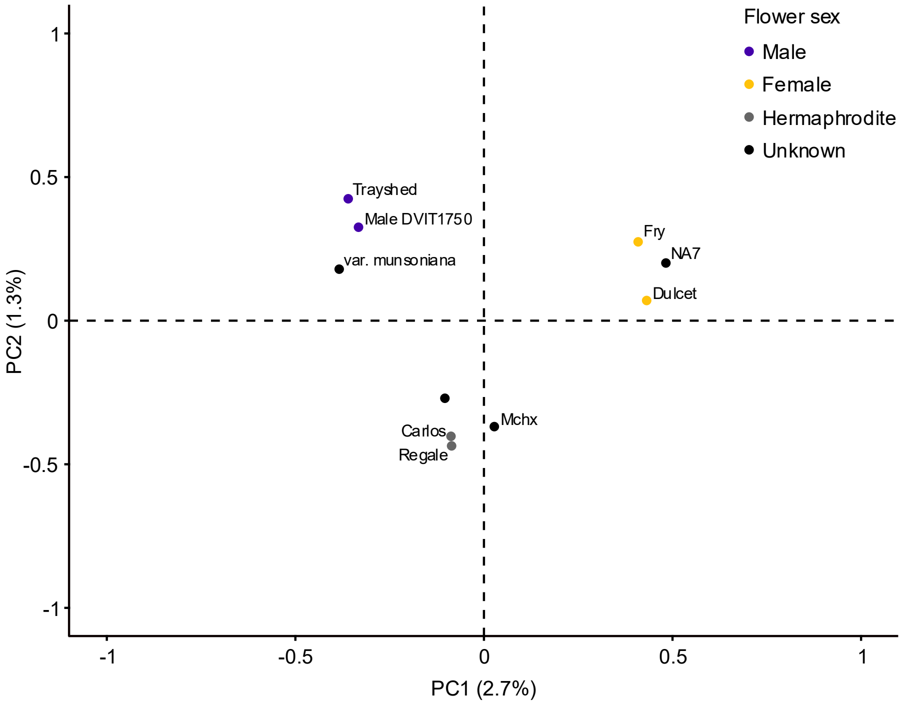

Supplementary Fig. 18. Principal components analysis of the SNPs comprised in the muscadine sex-determining region. Source data are provided as a Source Data file.

Supplementary references

1. Peng, D.-X. *et al*. Historical biogeography of *Tetrastigma* (Vitaceae): Insights into floristic exchange patterns between Asia and Australia. *Cladistics* **37**, 803–815 (2021).

2. Lewter, J. *et al*. High-density linkage maps and loci for berry color and flower sex in muscadine grape (*Vitis rotundifolia*). *TAG Theor. Appl. Genet. Theor. Angew. Genet.* **132**, 1571–1585 (2019).
