# Supplementary material for: Evolutionary conservation of the grape sex-determining region in angiosperms and emergence of dioecy in Vitaceae
Source: Nat Commun. 2025 Jul 1;16:6047. doi: 10.1038/s41467-025-61387-9 (PMC12215612; doi:10.1038/s41467-025-61387-9)
Supplement: Supplementary file 3 — Description of Additional Supplementary Files [file 41467_2025_61387_MOESM3_ESM.pdf]

Description of Additional Supplementary Files

File Name: Supplementary Data 1
Description: Information about the plant genomes used for the collinearity analyses.

File Name: Supplementary Data 2
Description: Annotation of the manually refined gene models (gff3).

File Name: Supplementary Data 3
Description: Information about the accessions sequenced in this study.

File Name: Supplementary Data 4
Description: Genome sequencing summary.

File Name: Supplementary Data 5
Description: Whole-genome assembly summary.

File Name: Supplementary Data 6
Description: Information about the short DNA-seq reads used in this study, and the status of the 8-bp INDEL in *VviINP1*.

File Name: Supplementary Data 7
Description: Summary of the intergenic repeats among the regions homologous to the *Vitis* sex-determining region. Source data are provided as a Source Data file.

File Name: Supplementary Data 8
Description: Transcription factors with different number of binding sites in M vs. F and H alleles of *VviYABBY3* promoter region.

File Name: Supplementary Data 9
Description: Sequences of the two LTRs of the two LTR retrotransposons identified between *VviPLATZ1* and *KASIII* in the M haplotypes of *M. rotundifolia*.

File Name: Supplementary Data 10
Description: Sequences of the ends of inversion used to evaluate genetic distances.

File Name: Supplementary Data 11
Description: Summary statistics of the RNA sequencing of ovaries and stamens from muscadine flowers.
