# Supplementary material for: Evolutionary conservation of the grape sex-determining region in angiosperms and emergence of dioecy in Vitaceae
Source: Nat Commun. 2025 Jul 1;16:6047. doi: 10.1038/s41467-025-61387-9 (PMC12215612; doi:10.1038/s41467-025-61387-9)
Supplement: Supplementary file 15 — Reporting Summary [file 41467_2025_61387_MOESM15_ESM.pdf]

nature portfolio

Corresponding author(s): Mélanie Massonnet, Dario Cantu

Last updated by author(s): Jun 10, 2025

Reporting Summary

Nature Portfolio wishes to improve the reproducibility of the work that we publish. This form provides structure for consistency and transparency in reporting. For further information on Nature Portfolio policies, see our Editorial Policies and the Editorial Policy Checklist.

Statistics

For all statistical analyses, confirm that the following items are present in the figure legend, table legend, main text, or Methods section.

n/a	Confirmed	
☐	☒	The exact sample size (*n*) for each experimental group/condition, given as a discrete number and unit of measurement
☐	☒	A statement on whether measurements were taken from distinct samples or whether the same sample was measured repeatedly
☐	☒	The statistical test(s) used AND whether they are one- or two-sided<br>*Only common tests should be described solely by name; describe more complex techniques in the Methods section.*
☒	☐	A description of all covariates tested
☒	☐	A description of any assumptions or corrections, such as tests of normality and adjustment for multiple comparisons
☐	☒	A full description of the statistical parameters including central tendency (e.g. means) or other basic estimates (e.g. regression coefficient) AND variation (e.g. standard deviation) or associated estimates of uncertainty (e.g. confidence intervals)
☐	☒	For null hypothesis testing, the test statistic (e.g. *F*, *t*, *r*) with confidence intervals, effect sizes, degrees of freedom and *P* value noted<br>*Give P values as exact values whenever suitable.*
☒	☐	For Bayesian analysis, information on the choice of priors and Markov chain Monte Carlo settings
☒	☐	For hierarchical and complex designs, identification of the appropriate level for tests and full reporting of outcomes
☒	☐	Estimates of effect sizes (e.g. Cohen's *d*, Pearson's *r*), indicating how they were calculated

Our web collection on statistics for biologists contains articles on many of the points above.

Software and code

Policy information about availability of computer code

Data collection	No software had been used for data collection.
Data analysis	FALCON-Unzip v.2017.06.28-18.01; hifiasm v.0.16.1-r374; RepeatMasker v.open-4.0.6; Augustus v.3.0.3; BLAT v.36x2; BUSCO v.3; EVidenceModeler v.1.1.1; Exonerate v.2.2.0; GeneMark-ES v.4.32; PASA v.2.1.0; SNAP v.2006-07-28; BUSCO v.5.4.7; RepeatModeler2 v.2.04; CD-HIT v4.6; miniprot v.0.4-r174-dirty; Cufflinks v.2.2.1; MUSCLE v.3.8.31; Gblocks v.91b; MEGAX; vcf2phylip.py v.2.0; DIAMOND blastp v2.0.13.151; MCScanX v.11.Nov.2013; GMAP v.2020-06-01; HaploSync v.1.0; seqtk v.1.2-r101-dirty; Trimmomatic v.0.36; Integrative Genomics Viewer v.2.4.14; Picard tools v.2.8; GATK v.4.2.2.0; vcftools v0.1.15; bcftools v.1.9; Plink v.1.90b5.2; Tomahawk v.beta-0.7.1; MUMmer v.4.0.0; PAML v.4.9; GenomeTools v.1.6.5; TFBSTools v.1.38; Salmon v.1.5.1; tximport v.1.20.0; GENESPACE v.1.3.1

For manuscripts utilizing custom algorithms or software that are central to the research but not yet described in published literature, software must be made available to editors and reviewers. We strongly encourage code deposition in a community repository (e.g. GitHub). See the Nature Portfolio guidelines for submitting code & software for further information.

Data

Policy information about availability of data

All manuscripts must include a data availability statement. This statement should provide the following information, where applicable:

- Accession codes, unique identifiers, or web links for publicly available datasets
- A description of any restrictions on data availability
- For clinical datasets or third party data, please ensure that the statement adheres to our policy

Sequencing data generated in this study have been deposited at NCBI under the BioProject PRJNA1151724 (https://www.ncbi.nlm.nih.gov/bioproject/PRJNA1151724). Genome sequences and gene annotation files have been deposited at Zenodo (https://zenodo.org/records/13362874).

Research involving human participants, their data, or biological material

Policy information about studies with human participants or human data. See also policy information about sex, gender (identity/presentation), and sexual orientation and race, ethnicity and racism.

Reporting on sex and gender	Not applicable.
Reporting on race, ethnicity, or other socially relevant groupings	Not applicable.
Population characteristics	Not applicable.
Recruitment	Not applicable.
Ethics oversight	Not applicable.

Note that full information on the approval of the study protocol must also be provided in the manuscript.

Field-specific reporting

Please select the one below that is the best fit for your research. If you are not sure, read the appropriate sections before making your selection.

☐ Life sciences ☐ Behavioural & social sciences ☒ Ecological, evolutionary & environmental sciences

For a reference copy of the document with all sections, see nature.com/documents/nr-reporting-summary-flat.pdf

Ecological, evolutionary & environmental sciences study design

All studies must disclose on these points even when the disclosure is negative.

Study description	This study explores the evolutionary conservation of the Vitis sex-determining region (SDR) across angiosperms, the evolution of this locus in Vitaceae, and the emergence of dioecy in the Vitis/Muscadinia lineage. We analyzed the conservation of the Vitis SDR across 56 plant genomes and found collinearity in all 43 angiosperms, but not in non-flowering plants. We further explored the SDR conservation within the Vitaceae family by long-read sequencing and haplotype phasing of eight Vitaceae individuals: two Muscadinia rotundifolia (one female and one male), Parthenocissus quinquefolia, two Ampelopsis species (A. aconitifolia and A. vitifolia), two Cissus species (C. amazonica and C. gongylodes), Tetrastigma voinieranum, as well as the outgroup Leea coccinea. Using short DNA-sequencing reads from 13 Tetrastigma species, no recombination suppression was found in the dioecious Tetrastigma, suggesting a different sex determination mechanism. In Muscadinia rotundifolia, linkage disequilibrium analysis using the short DNA-sequencing reads from ten individuals, comparison of six SDR haplotypes (2 M and 4 F), and gene expression quantification of the F and M alleles of VviYABBY3 in ovaries and stamens from three individuals (three bioreplicates per condition), showed that muscadine grapes have similar SDR boundaries and candidate sex-determining genes as Vitis. Finally, visualization of the alignment of DNA-seq short reads from 159 Vitaceae accessions, showed that the candidate male-sterility mutation was absent in all accessions, including the two other Viteae, Ampelocissus and Pterisanthes. This suggests that the candidate male-sterility mutation is specific to the Vitis and Muscadinia genera.
Research sample	For genome sequencing, young leaves were collected from 17 accessions: two M. rotundifolia individuals (Fry and DVIT1750), six Ampelopsis individuals representing four species, six Cissus species, P. quinquefolia DVIT2400, T. voinieranum, and L. coccinea 1464. For RNA-sequencing, inflorescences from M. rotundifolia Fry (female), M. rotundifolia Trayshed (male), and M. rotundifolia DVIT1750 (male), were collected at full flowering with 50% caps off (E-L 23 (Coombe, 1995)). Ovaries and stamens from cap-off flowers were then sampled to represent three biological replicates per accession.
Sampling strategy	Flower ovaries and stamens were sampled from three inflorescences, separately, representing three biological replicates.
Data collection	Sequencings were performed at DNA Technology Core Facility, University of California, Davis.
Timing and spatial scale	Leaves for DNA extraction were collected from plants at the University of California Davis (Davis, CA, USA), the USDA National clonal germplasm repository, Davis (Winters, CA, USA), and the USDA New York State Agricultural Experimental Station (Geneva, NY, USA) in

	the last one to four years.<br>For RNA-seq, inflorescences were collected in June 2023 from vines at the University of California Davis (Davis, CA, USA).
Data exclusions	No data were excluded.
Reproducibility	Not applicable.
Randomization	Not applicable.
Blinding	Not applicable.

Did the study involve field work? ☒ Yes ☐ No

Field work, collection and transport

Field conditions	Not applicable.
Location	Sampling was performed from plants located at the University of California Davis (Davis, CA, USA; 38°31'44.9"N 121°47'26.4"W), the USDA National clonal germplasm repository, Davis (Winters, CA, USA; 38°30'16.5"N 121°58'50.2"W), and the USDA New York State Agricultural Experimental Station (Geneva, NY, USA; 42°52'37.5"N 77°00'26.3"W).
Access & import/export	Plants from the USDA National clonal germplasm repository, Davis were accessed through the courtesy of Claire Heinitz (National Clonal Germplasm, USDA-ARS). Leea coccinea was accessed by courtesy of Ernesto Sandoval (University of California Davis).
Disturbance	No disturbance was caused by the study.

Reporting for specific materials, systems and methods

We require information from authors about some types of materials, experimental systems and methods used in many studies. Here, indicate whether each material, system or method listed is relevant to your study. If you are not sure if a list item applies to your research, read the appropriate section before selecting a response.

Materials & experimental systems

n/a	Involved in the study
☒	☐ Antibodies
☒	☐ Eukaryotic cell lines
☒	☐ Palaeontology and archaeology
☒	☐ Animals and other organisms
☒	☐ Clinical data
☒	☐ Dual use research of concern
☐	☒ Plants

Methods

n/a	Involved in the study
☒	☐ ChIP-seq
☒	☐ Flow cytometry
☒	☐ MRI-based neuroimaging

Dual use research of concern

Policy information about dual use research of concern

Hazards

Could the accidental, deliberate or reckless misuse of agents or technologies generated in the work, or the application of information presented in the manuscript, pose a threat to:

No	Yes	
☒	☐	Public health
☒	☐	National security
☒	☐	Crops and/or livestock
☒	☐	Ecosystems
☒	☐	Any other significant area

Experiments of concern

Does the work involve any of these experiments of concern:

No	Yes	
☒	☐	Demonstrate how to render a vaccine ineffective
☒	☐	Confer resistance to therapeutically useful antibiotics or antiviral agents
☒	☐	Enhance the virulence of a pathogen or render a nonpathogen virulent
☒	☐	Increase transmissibility of a pathogen
☒	☐	Alter the host range of a pathogen
☒	☐	Enable evasion of diagnostic/detection modalities
☒	☐	Enable the weaponization of a biological agent or toxin
☒	☐	Any other potentially harmful combination of experiments and agents

Plants

Seed stocks	Plant material was sampled from plants located at the University of California Davis (Davis, CA, USA), the USDA National clonal germplasm repository, Davis (Winters, CA, USA), and the USDA New York State Agricultural Experimental Station (Geneva, NY, USA).
Novel plant genotypes	Not applicable.
Authentication	Not applicable.
